# Accessing health information during the COVID-19 pandemic: the experience of NHS maternity service users

**DOI:** 10.1186/s12884-023-06160-w

**Published:** 2023-12-11

**Authors:** Rushvini Ambihaipahan, Georgia Chisnall, Cecilia Vindrola-Padros, Lucy Irvine

**Affiliations:** 1 University College London, Institute of Global Health, London, UK; 2 Department of Targeted Intervention, UCL, London, UK

**Keywords:** COVID-19 pandemic, Pregnancy, Women, Antenatal, Vaccine

## Abstract

**Background:**

The COVID-19 pandemic caused various disruptions to NHS maternity services in England. Changes were made to antenatal and postnatal care and the way that information was shared with maternity service users during these times. Fewer face-to-face appointments, increased virtual appointments and changes in guidance about the suitability of the COVID-19 vaccine without appropriate information sharing and evidence caused concern.

**Methods:**

This study took a blended inductive-deductive approach to secondary data analysis using a population subset of 16 from a wider study that sought to understand the impact of COVID-19 on maternity services in England. Participants of this study were aged 28–44 and gave birth using NHS maternity services in England. The data were collected and coded using Rapid Analysis Procedure sheets, which generated key themes, which are used here to structure the results.

**Results:**

Four main themes were generated from the analysis: 1) service restrictions to antenatal and postnatal appointments 2) access to information and changes to antenatal and postnatal care 3) inconsistencies in the implementation of government and NHS policy and 4) limited information about COVID-19 vaccine provided by NHS trusts and hesitancy in vaccine acceptance.

**Conclusion:**

Participants experienced poor communication that affected their understanding of maternity service changes and there was limited general and maternal health information provided. Vaccine information was also inadequate, and participants expressed a desire for clearer guidance. The UK Government, Royal College of Obstetricians and Gynaecologists, and NHS must collaborate with maternity service users to ensure that there are evidence-based guidelines and policies that can be understood and standardised across all NHS maternity trusts.

## Introduction

COVID-19 is a contagious disease spread by the SARS-COV-2 coronavirus that first emerged in humans in 2019 [1]. In the first two years of the pandemic there were over 450 million cases of COVID-19 reported worldwide [1]. Although there have been other strains of coronavirus, SARS-COV-2 had previously not been recognised and identified in humans and so posed several challenges to the UK National Health Service (NHS). The first national lockdown was initiated in late March 2020, during this time all non-essential businesses were closed, and people were advised to stay at home [2]. People were permitted to leave for essential purposes only including to buy food or seek emergency medical attention [2]. Maternity services were included within emergency services during the lockdown.

Nonetheless, maternity care was affected by the COVID-19 pandemic and saw many changes to service provision around the globe. In the NHS in England, maternity services were reduced due to staff shortages and to reduce the risk of transmission in clinical settings, whilst meeting the minimum levels that were required to keep women and their babies safe. Changes included a reduction in face-to-face antenatal and postnatal contact, closure of midwifery led units, suspension of homebirth teams and restrictions to birth partner and visitor attendance [3]. These changes varied considerably between trusts (locations of healthcare provision, including hospitals), and were often poorly communicated to service users. This meant that in many cases users had to proactively search for health information and information about changes to services in their area. This finding on limited access to health information has been cited in studies by Meaney et al. [4] and Riley et al. [5].

Although restrictions including lockdowns and social distancing measures placed on people during the COVID-19 pandemic were deemed essential by the UK government, it is important to understand how this affected maternity service users and the effect that this had on their lived experiences during the pandemic. Immediate concerns have already been shared by a charity Birthrights, who called for “robust national guidance from NHS England” shortly after the first lockdown was instated [6].

This study aimed to understand the impact the COVID-19 pandemic had on maternity service users’ access to information provided by the NHS. Qualitative data from semi-structured interviews were used to understand how the women participating in the study felt about the way information was communicated to them focusing on service changes, health, and vaccine information. The maternity service users commented on their experiences of antenatal care including booking their appointments and ultrasound scans, experiences during labour, and delivery and on the postnatal ward.

Prior to this there were clear gaps in the literature regarding women’s access to general and maternal health information during and after their pregnancy. Although there was a body of literature surrounding the COVID-19 vaccine in pregnancy, there was only one study published by Skirrow et al. [7] that is based in the UK and evaluates women’s views towards accepting the vaccine during and after pregnancy and none reviewing where women sought vaccine information from. Aside from the studies by Karavadra et al. [8] and Sanders and Blaylock [3], which were both conducted in the United Kingdom and looked generally at the experiences of pregnant women during the pandemic, it appeared that there were no other qualitative studies conducted on this group and none with a focus on access to maternal health and vaccine information.

### Terminology

All the participants identified as ‘women’ and the term is used in this study whilst acknowledging that not all people who become pregnant and give birth identify as women.

### COVID-19 in pregnancy

Early in the pandemic, it was unclear how COVID-19 affected the health of pregnant women and their babies. Government guidance recommended that all pregnant women should follow stringent social distance guidance and they were placed in the same risk category as people aged over seventy and those with a weakened immune system [9]. Pregnant women who were clinically vulnerable were advised to “shield” [9]. ‘Shielding’ was a term used by the UK Government to protect people who were at the highest risk of being hospitalised by COVID-19, they were advised not to leave their homes and within their home to minimise time spent with others and to avoid using shared spaces when others were present [10].

In December 2020, the UK Government identified all pregnant women as being at a higher risk of severe illness if they were infected with SARS-COV-2 and then developed COVID-19 [11]. The Royal College of Obstetrics and Gynaecology (RCOG) subsequently advised in 2020 that pregnant women should maintain social distancing and self-isolate to lower their risk of exposure [11]. Updated RCOG guidelines in 2022 suggest that pregnant women with no co-morbidities are not any more or less likely to contract the infection than the general population but pregnant women with co-morbidities are at an increased risk of contracting the virus [12, 13]. Pregnant women may also be at increased risk of severe illness from COVID-19 compared to non-pregnant women, particularly in the third trimester [12, 13].

### Maternity care

As the pandemic evolved, guidance from the RCOG in late 2020 stated that the National Institute for Health and Care Excellence (NICE) schedule of antenatal appointments should be maintained which included eight antenatal appointments and at least six of these held in-person [14]. This was not always followed in its entirety by NHS trusts that delivered maternity care. A study investigating local modifications to maternity care during the COVID-19 pandemic suggested that there were changes to the nature and frequency of antenatal care appointments [15]. The study compared local modifications by hospital sites to existing national pandemic frameworks issued by the RCOG, Royal College of Medicine, and the NHS. Flaherty et al. [16] described a move towards telehealth and remote antenatal and postnatal appointments in addition to reduced or altered postnatal support. Jardine et al. [15] stated that two-thirds of units reported a reduction in antenatal appointments and that almost all services had some remote appointments such as telephone consultations. Respondent units modified postnatal services including reducing routine post-natal contact for low-risk women and using telephone or videoconferencing for virtual appointments [15]. Another study reviewing women’s perceptions of COVID-19 and their healthcare experiences found that participants understood the need for virtual appointments but felt that it was impersonal [8]. Flaherty et al. [16] highlighted that for many women who gave birth during the pandemic most did so in a system that did not allow their birth partner to attend antenatal and postnatal appointments. In many hospital trusts, birth partners had restrictions placed upon them and were limited to only attending active labour [16]. Although the long-term effects that these restrictions had on parental bonding and postnatal mental health are yet to be studied, changes to antenatal, labour, and postnatal care were often sudden and would have been distressing for women and their birth partners.

### Access to health information

Access to good quality health information was challenging during the pandemic and there was a rise in misinformation associated with political and economic instability [17]. During the pandemic the UK Government provided guidance to the public and developed a response plan with the medical experts from Scientific Advisory Group for Emergencies (SAGE) [18]. The RCOG are a professional medication association that worked closely with the government and the UK Health Surveillance Agency to advise and produce guidelines on women’s health [19]. NHS England are the national body that set priorities for healthcare which further filters into individual NHS trusts who apply them [20].

At the start of the pandemic there was no formal guidance issued by the government and the RCOG on where pregnant women should obtain their health information from. This led to women obtaining health information from non-reputable websites which put them at risk [21]. The risks of misinformation and poor quality information were discussed by the Royal College of Midwives [21] in respect to the decision surrounding taking the COVID-19 vaccine. These risks included harm to the women themselves, stillbirth, preterm delivery, and the need for more interventions at birth [21].

Lack of usual access to healthcare professionals via face-to-face appointments also made it more challenging for women to discuss concerns with specialists and many women relied upon the expertise of family members or friends who had experienced pregnancy and childbirth in place of professional medical advice [22]. Sanders and Blaylock [3] reported that many participants had to review changing guidelines by themselves and that there was a lack of direct communication from NHS organisations and midwives.

### The COVID-19 vaccine

Throughout the pandemic there was varied information and advice from the government and NHS trusts about whether the COVID-19 vaccine should be taken by pregnant women. Pregnant women were not included in the initial Pfizer and BioNTech COVID-19 vaccination trials [7, 23] which resulted in uncertainty regarding whether the vaccine was suitable for pregnant women. Early guidance from the UK’s joint committee on Vaccination and Immunisation (JCVI) stated that women should not be offered the COVID-19 vaccine due to the lack of data on its safety during pregnancy [7]. At the end of December 2020, pregnant women who were frontline workers and pregnant women with other risk factors for severe COVID-19 infection were offered the vaccine [7]. Advice changed as new evidence emerged and current guidance strongly recommends two doses of the COVID-19 vaccines and a booster in pregnancy [12]. The JCVI advises that all pregnant women in the UK should be offered the Comirnaty/Pfizer BioNTech or Moderna Spikevax mRNA vaccine where available as the data from these vaccines have not raised safety concerns. Women who have already had one dose of the Oxford-AstraZeneca are advised to complete vaccination with the second dose of Oxford-AstraZeneca, there have been no reported concerns with this vaccine in pregnancy but there is less published data with this vaccine [13]. A systemic review and meta-analysis which reviewed the administration of COVID-19 vaccination during pregnancy showed no significant association between the COVID-19 vaccination during pregnancy and increased adverse pregnancy outcomes [24].

Inconsistent advice surrounding the COVID-19 vaccine has been linked to varied vaccine acceptance. A UK survey of women who were pregnant from March 2020 to October 2020 found that vaccine acceptability was highest when women were not pregnant and there were concerns over the speed of the COVID-19 vaccine development, lack of information on safety and side effect profile, and mistrust of health advice [7].

## Methods

A secondary analysis was performed on a population subset of a wider study that sought to understand the impact of COVID-19 on maternity services in England. The original study was conducted by LCI and GC. The aim of the original study was to understand how women using NHS services during pregnancy and labour experienced care during the COVID-19 pandemic in the UK, and their perceptions of birthplace safety. This secondary analysis explores concepts which were beyond the scope of the original analysis with a focus on factors associated with access to health information, and participants views on the COVID-19 vaccine.

### Participant recruitment and sampling

Of the forty-six interviews available five interviews were discounted from the present analysis as they did not include discussion with the participants about the COVID-19 vaccine. The remaining forty-one interview transcripts were then reviewed. Data saturation was reached after sixteen interviews, where the authors noted that the data did not provide anything new, and the same themes were emerging from each transcript. Hence, this paper consists of secondary data analysis of a subset of 16 interviews of a larger dataset of 46 interviews with women aged between 18–45 who delivered their babies between 1^st^ March 2020 and 1^st^ March 2021 using NHS services in England, and who had low risk pregnancies. In the original study participants were recruited using social media adverts and participants who made contact and were eligible for selection were sent the information sheet and consent form.

### Data collection procedures

Data collection took place as part of the original study, during which interviews were conducted via WhatsApp and Microsoft Teams. Interviews were recorded with a separate voice recorder and transcribed using NVivo transcription software. Interviews were held between the 25^th^ March and 13^th^ May 2021 and lasted between 30 to 75 min.

### Data analysis

Based on emerging research and anecdotal evidence at the time of study design, it was hypothesised that there would be a negative impact on women’s experiences of maternity care, but it was unclear in what ways. Resultantly, a blended inductive-deductive approach was selected, where the key themes – namely the most frequently reported negative impacts on care –were identified from the data itself through coding and thematic analysis. Thematic analysis was used to analyse patterns in qualitative data and was used to generate the themes in this study [25]. Thematic analysis involves familiarisation with the data (by listening to the interviews), systematic coding of the data (capturing key features of the data), generation of themes (through looking at the codes to identify overlap) and review of the themes with the coded data and applying it to the study question [25].

A team-based approach to data analysis was facilitated using RREAL (RAP) Sheets [26]. RREAL Sheets are working documents created on a study-by-study basis which are used to analyse data on an ongoing basis throughout the data collection period and build upon the well-established use of table-based methods in qualitative research such as framework analysis [27]. RREAL Sheets are designed as a table with two columns. The first column is composed of pre-established categories of interest identified at the start of the study and the second column contains focused annotations made by the researchers for each category. For this study, pre-identified categories were guided by the devised interview topic guide. During interviews researchers took notes in real-time using the RREAL Sheet template as a template (hence a sheet was completed *per participant*). Following the interview, researchers listened to the interview recording and ensured that all points were accurately represented in the *per participant* RREAL Sheet.

Interviews were transcribed using NVivo audio to script software, but these tended to contain errors so were used as rough guides to locate key parts of the interview. Researchers then re-listened to interview recordings and added notes and quotes to the RREAL sheets, including transcribing what were deemed to be key excerpts of the interview. As more interviews were carried out, it was possible to identify common repeating themes within the separate sections of the RREAL sheets. All authors met regularly to discuss findings (i.e., themes) and to facilitate ongoing team-based analysis of the RREAL sheets. A separate RAP sheet was created by RA specifically for the secondary data analysis, with the first column populated with categories of interest focussed on access to health information, communication from health professionals, and the COVID-19 vaccine.

### Ethics

Access to the dataset was needed and ethical approval for this was obtained from the University College London Research Ethics Committee (Project ID: 19863/001). The participants involved in the primary data collection were aware that their interviews would be used for secondary research looking at maternity care and consented to this.

### Positionality

RA works as a Paediatric doctor within the NHS. LCI is a lecturer at the Institute of Global Health at University College London. RA worked as a paediatric doctor at a large neonatal unit in Scotland in 2020 and was involved antenatal counselling for women and their birth partners during the COVID-19 pandemic. RA is interested in the link between public health policy, the patient experience and quality of care received. LCI is a social scientist working in maternal health and believes that qualitative data provide compelling evidence of the impact of policy decisions on people’s lived experience and their health, regardless of its measurability in health outcome data. LCI believes that high quality maternity care is determined by the extent to which women and their families feel informed and empowered to make decisions about their care. GC is a qualitative social scientist with a background in heath psychology. GC works in health service research and aligns with the beliefs outlined by LI.

## Results

Table 1 shows the key characteristics for the participants of the study including information on age, place of delivery, mode of delivery, socio-economic status, and ethnicity.

**Table 1:** Key characteristics for the participants of the study

*Age*	
Mean	34.1 years
Median	34 years
Mode	33,34 years
Youngest	28 years
Oldest	44 years
*Place of delivery*	
Gloucestershire	*n* = 1
London	*n* = 12
Milton Keynes	*n* = 1
Surrey	*n* = 1
Worcester	*n* = 1
*Mode of delivery*	
Assisted Vaginal Delivery	*n* = 7
Vaginal Delivery	*n* = 4
Emergency C-Section	*n* = 4
Elective C-Section	*n* = 1
*Socio-economic Classification*^*a*^	
Higher managerial, administrative, professional occupations	*n* = 2
Intermediate occupations	*n* = 13
Routine and manual occupations	*n* = 0
Never worked or in long-term unemployment	*n* = 1
*Ethnicity*	
White British	*n* = 13
Mixed Ethnicity/White Other	*n* = 3

Participants who had never worked or were in long-term unemployment were placed in a fourth category

^a^Participants have been grouped into three categories as per the National Statistics Socio-economic Classification; the official socio-economic classification used in the United Kingdom [28]

Four main themes were identified from the analysis, an overview is provided in Table 2. These themes are presented in no particular order. While these themes are independent it is conceptually relevant that they most likely intersect in relation to women’s’ experiences of maternity care. For instance, a virtual mode of appointment (theme 1) may be associated with reduced access to information (theme 2), and increased COVID-19 vaccination hesitancy (theme 4).

**Table 2:** Overview and definition of identified themes

**Theme Title**	**Definition**
Theme 1: Impact of service restrictions on antenatal and postnatal care	**This theme captured the participants engagement with maternity services and the impact that this had on their care**
Theme 2: Access to information and changes to antenatal and postnatal care	This theme considered the experiences of the participants while accessing maternity and health information and some of the challenges they faced
Theme 3: Inconsistencies in the implementation of government and NHS policy	**This theme captured the participants experiences of government and NHS policy and how it affected their care**
Theme 4: Limited information about COVID-19 vaccine provided by NHS trusts and hesitancy in vaccine acceptance	**This theme considered processes involved in the participants decisions to take the COVID-19 vaccine**

### Key themes

#### Theme 1: impact of service restrictions on antenatal and postnatal care

This theme captured participants engagement with maternity services both during their pregnancy and in the postnatal period. The maternity service users included in this study reported that both their antenatal and postnatal care were affected by service restrictions. Analysis of the data suggested variation in the experiences of participants in terms of their antenatal and postnatal care appointments, with some women continuing to have face-to-face appointments, some completely virtual and for many a combination of both. The participants described being aware of and feeling dissatisfied by the differences in service provision between NHS trusts.

*“You don’t want to feel like it’s luck of the draw. You want to feel like everything is standardised for every pregnant woman”. Participant 07*

Common changes to antenatal care included making the initial booking appointment a telephone appointment, disallowing birth partners to attend ultrasound scans and rescheduling existing appointments to a later date. These changes left women feeling unsupported during their pregnancy and their partners feeling excluded from the experience. Three of the participants opted to supplement routine NHS ultrasound scans with private ultrasound scans with the most common reason being that women felt that their birth partners were missing out on the opportunity to be involved as well as wanting health information to be shared with another person.

*“He only came to the very first midwife appointment and then after that I did everything on my own …if I was able to have my partner with me, two things would have happened, I would have felt more back up… and he would have been much more integrated and involved in the process” Participant 12*

One participant who was sixteen weeks pregnant when lockdown began explained that her early antenatal scans occurred as normal but at the scan she was informed of a complication and required a repeat scan at sixteen weeks, which then had to be rearranged due to concerns about COVID-19 safety.

*“Just as this scan was coming up, people were saying partners aren’t going to be going to scans… That scan was delayed a week. I had got it in my mind the date for this scan that would find out if it was a genetic condition that the baby might have, and they pushed it back a week and…it was very confusing” Participant 08*

Another cause of concern for the participants was that due to sickness, staff shortage and redeployment many women did not see the same midwife throughout their pregnancy and described feeling an absence of continuity of care.

*“Depending on the trust that you’re with you don’t see the same midwife often and I think during COVID it would be better if you did…that continuity of care would be appreciated even more during COVID” participant 07*

Service restrictions did have an impact on the care that the participants received during labour and delivery. The possible absence of birth partners from all or parts of the delivery was of most concern before women gave birth and caused immense distress when for many this was realised.

*“Then my husband was kicked out… I had no time to consider what just had just happened…I hadn’t considered what managing after a c-section would be like without my husband…we hadn’t even looked at each other to say- well done on having this baby…” Participant 11*

*“So, I had 4 or 5 contractions in the street walking from the car to the reception area…the security guard was like- no no no, you can’t come in, to my partner which was a surprise because I thought partners were allowed to come in for the labour. I was clearly in advanced labour…because I was having these contractions, I just had to comply” Participant 12*

Participants described finding the postnatal period particularly challenging. Many attributed this to rules stating that birth partners were not allowed on the postnatal ward or that they had allocated time slots.

*“We were moved into a private room… maybe a couple of hours after that a midwife or someone popped in and sternly said you need to go to [partner] you can’t be here… it was the middle of the night…I started feeling quite nervous to be left alone” Participant 12*

Participants also reflected on the strain that the restrictions to maternity services had on them. They described facing added pressures including needing to ask their midwives relevant questions and then sharing this information with their birth partners and the distress caused by rescheduling appointments and having to wait longer to be given crucial information. As discussed, service restrictions have had a significant impact on the birth experience but the effect on acute and long term mental health which is highlighted by the reflections above should also be considered.

#### Theme 2: access to information and changes to antenatal and postnatal care

This theme captured the experiences of participants while accessing maternity and health information and some of the challenges they faced.

Overall, most participants did believe that changes to antenatal and postnatal care were communicated to them, but with varying degrees of accuracy and success. Changes were made at short notice, and it was often left up to women to seek out information themselves. The most common way that service changes were communicated to the participants was through updates to hospital websites and their associated Facebook and Twitter pages.

*“I think I saw that partners weren’t allowed at the scans from looking at the [hospital] Twitter page. We weren’t really contacted by the midwives and updated on what was happening, which was quite confusing. I did spend a lot of time like most days just obsessively checking Googling “[hospital] maternity” …to see what was changing” Participant 10*

Many of the participants commented that even when information was shared with them it was often of poor quality and difficult to interpret.

*“Very vague really. I mean when we were doing the scans, I pretty much phoned the hospital the day before to say will my partner be able to come in” Participant 15*

The responses highlighted a clear lack of communication affecting maternity service users. The poor communication involved both antenatal and postnatal services and it was evident from the responses that not all members of the maternity team were able to provide up to date information for women. Many of the participants described needing to seek clarification about aspects of their care from several members of the clinical team and reported that they were not always given the same response. This left them feeling uncertain and concerned about their quality of care.

*“I think was surprised because they’re so good at communication externally, the communication lacked when I was there…no one really knew, people were always asking someone else” Participant 11*

When women were asked about where they accessed general and maternal health information eleven participants described using NHS trust websites and their associated sites (Facebook, Instagram, Twitter, and dedicated hospital COVID-19 phone lines), making the hospital trusts and their various channels of communication the most popular way to access information. The RCOG website closely followed, as did the NHS website and support groups (local mum’s groups and WhatsApp National Childbirth Trust groups). Many participants reported that the health information provided on the government website (gov.uk) was particularly challenging to interpret and seen as unreliable.

*“We were looking at gov.uk which we found there was just a lot of information…it was hard to find exactly what we were looking for,,,. Some places were saying a blanket statement that everyone was high risk…other sources were saying that pregnant women are not at higher risk if they got COVID than someone who was not pregnant” Participant 03*

*“I knew that no one knew exactly… I was very untrusting of anything that came out of the cabinet office or the government or the DOH (Department of Health)” Participant 04*

Participants also reported feeling unable to ask their midwife questions due to time constraints or felt that their questions were insignificant in the context of the pandemic. The National Childbirth Trust is one example of a support group that offered antenatal courses, information and local activities for expecting and new mothers and was mentioned by several of the participants as a useful organisation particularly during the COVID-19 pandemic [29].

Although a variety of sources were used it was evident that there was confusion surrounding which sources were accurate and reliable. All the women in the study used more than one source to access general and maternity information but continued to remain unsure about the risk of COVID-19 to their baby and themselves.

#### Theme 3: inconsistencies in the implementation of government and NHS policy

A central theme that emerged was the inconsistency in implementation of government and NHS policy by NHS Trusts and the effect that this had on women and their birth partners. Many of the participants commented that the care that they received from one NHS trust was significantly different to the care that their friends received at another NHS trust.

*“That was another thing that was discombobulating, was that when you’re talking to different people, everyone’s having different experiences, it doesn’t feel like there is a unified message about what’s safe and why some people are getting a level of care that others aren’t” Participant 09*

*“There didn’t seem to be a reason for some of this stuff especially not letting partners in… Lack of consistency between trusts… this was the thing that felt most upsetting” Participant 09*

Another participant wondered why guidance issued by the NHS England had not been implemented across all NHS trusts. Women were also unsure about following early governmental advice regarding shielding and suggested that this uncertainty stemmed from rapidly changing advice.

*“I’ve read that…they’ve issued statements saying that pregnant women should have somebody with them, but it still seems to not be completely implemented. …Why is it still not consistent across all hospitals and what can we do to try and make it so? Participant 13*

*“The amount of times everything changed…and that it was worded differently. And then they were like adding things and taking things away… So, the level of consistency was shocking, shockingly bad.” Participant 03*

Participants reported that restrictions placed on birth partners attending ultrasound appointments, labour and delivery and postnatal appointments did not always correspond with the stage of the pandemic and the number of cases of COVID-19 in their local area.

*“There were so many rules saying my partner was not allowed to come to the scans and the midwife appointments. Even all through the summer when the COVID numbers were really low… and then when we saw the health visitor…I asked can my husband come in? And they were like that’s fine…which was good but frustrating because at that point the COVID numbers were really high, and it seemed like no big deal. So why had he not been able to come to those previous appointments?” Participant 15*

*“The main thing for me is that partners should be present throughout the whole process.*

*… The thing that made me really sad was that we were being denied that, while it was still ok for people to go to restaurants and pubs…but we weren’t allowed to be one to one with the person who is most important to us at that moment” Participant 03*

Inconsistencies between government and NHS policy made it hard for women to trust the advice that they were given and contributed to the difficulties participants faced in accessing accurate health information. Participants described being torn between following government and NHS advice because it was the safest option and understanding where the evidence behind many of the decisions made came from.

#### Theme 4: limited information about COVID-19 vaccine provided by NHS trusts and hesitancy in vaccine acceptance

This theme captured the processes involved in the participant’s decision to take the COVID-19 vaccine. The interview data suggested that women were affected by the initial mixed messages about the COVID-19 vaccine that were conveyed by the government, NHS and the RCOG, and many expressed hesitancy in taking the vaccine, especially while they were breastfeeding.

The study showed that most women obtained information about the COVID-19 vaccine through independent research and very few women were provided information by their NHS trust. Women were also uncertain about what sources they should be using to find out this information. Most concerning to women was the exclusion of pregnant women from vaccine clinical trials and its safety for mothers who are breastfeeding. Women reported obtaining vaccine information from the NHS website, RCOG, CDC, the WHO and through social media.

*“I got my jab on March 30*^*th*^*…I have mixed feelings because I don’t like that there a lack of data for breastfeeding women… I made the decision that the benefits outweighed the risk for me, and I did take it…” Participant 13*

*“I’m in two minds about what to do about it… I’ve not been called yet and as and when I get called; I think I’ll make a decision then” Participant 05*

The above excerpts show that participants were affected by the initial mixed messages surrounding the COVID-19 vaccine and were hesitant to accept it based on the limited information available at the time. Most of the participants wanted to wait until they stopped breastfeeding before having the vaccine for this reason.

## Discussion

The COVID-19 pandemic created many challenges for the provision of maternity care in England. This study found that women were affected by maternity serviced restrictions and limited access to health information. It was clear that their care during and after pregnancy was affected by inconsistent implementation of government policies. This had ramifications for their pregnancy experience and had a significant impact on not only their perceptions of the care that they received but also their mental health. Lack of clear and concise information provided by both NHS trusts and the government impacted on decision making and led to hesitancy on whether to accept the vaccine.

This study contributes to existing studies on maternity service users’ experiences during the pandemic, whilst also providing new insights into their access to health and COVID-19 vaccine information [7, 8, 16]. Expectant and new mothers have also had to make decisions that, prior to the pandemic, they would not have been expected to make. This includes the decision to vaccinate and whether to follow shielding advice.

Access to general and maternal health information during the pandemic was a challenge for the participants of this study. Women used a variety of sources to access information but often reported feeling confused by what they were reading or were mistrusting of it. Information from the government was often unclear and led to many of the participants making independent health-related decisions, this applied mainly to shielding and whether to let relatives visit during the postnatal period.

The variation in the implementation of government and NHS policy caused concern and many participants reported differences in the care provided between trusts. Similar findings were found by Jardine et al. [15] in the study of modifications to standard maternity care. Birthrights, a UK charity, have provided further evidence of this through correspondence with various NHS trusts about decisions made to maternity care [30]. This showed discrepancies to service provision, allowance of birth partners, and visiting policies between NHS trusts [30].

Another important finding from this study was the role of support groups, friends who were also pregnant or had recently given birth, and relatives who had medical knowledge in providing participants with health information. This was often in place of formal obstetric and midwifery advice. A qualitative study (*n* = 23) of women who gave birth during the pandemic in London reported similar findings regarding their reliance on informal support groups during the COVID-19 pandemic [22]. While it is important for women to have support from family, friends, and support groups it is still essential for women to have their questions and concerns addressed by their own care providers or by government public health bodies.

In general, participants accepted that maternity services needed to change because of the pandemic, and many were sympathetic to the effect that COVID-19 had on healthcare workers and the NHS. However, the communication of maternity service changes was often felt to be lacking. Several participants reported being given a limited amount of information from their midwife or being given information at very short notice. Examples of this included not being informed about whether their birth partners would be allowed at antenatal scans and birth partners being asked to leave very suddenly when women were transferred to the postnatal ward. Although it is important to recognise that the COVID-19 pandemic was novel and was rapidly changing during the time that these participants required maternity care, good communication is vital in clinical care and as highlighted by this study can have lasting effect on how patients view their experience. A lack of maternal health information was also noted in the study by Karavadra [8].

It was clear that there was limited information provided about the COVID-19 vaccine by NHS trusts and most advice given contradicted earlier advice to not take the vaccine which resulted in suspicion. Women were willing to independently search for information about the vaccine, but most were hesitant to take the vaccine during pregnancy. These findings are comparable to those reported by Skirrow et al. [7]. The findings of this study suggested that participants were concerned about the exclusion of pregnant women from clinical trials, but none of the participants commented on whether they would take part in a clinical trial. It is clear that women require safety information and clear communication before deciding to accept a vaccine, findings that have also been noted by Skirrow et al. [7].

### Strengths and limitations

The main strength of this study was that it involved the detailed analysis of sixteen interviews and examined a broad range of topics related to the use of NHS maternity services during the COVID-19 pandemic. A flexible interview technique was achieved by asking open ended questions and giving participants the time to share their experiences, which was important given the sensitivity of the topic. The interviews were conducted online which was convenient for new mothers and enabled them to comply with social distancing advice. Each interview ranged between 30 and 75 min which the interviewers felt provided sufficient time to gain a comprehensive understanding of the experiences faced, however, maternity service users may have felt time restricted in what they were able to discuss due to the limited time available.

In this selected sample over 81% of participants described their ethnicity as White British. This is in line with 80.5% that was reported in the 2011 Census as the majority ethnic group [31]. However, most of the participants in this study were based in London where in 2011, 40.2% identified as Asian, Black, Mixed or Other ethnic groups [32], suggesting a low ethnic minority representation. The study would have benefited from a more diverse group of participants, particularly given COVID-19 disproportionately affects Black, Asian and minority ethnic groups through a combination of physical co-morbidities and possible health and social inequalities [33].

Most of the participants worked in higher managerial, professional occupations and in intermediate occupations. This may represent self-selection bias where these participants had more time to take part in the study and were more comfortable in sharing and critiquing their experience. Future studies need to incorporate the views of pregnant women in routine and manual occupations and women who were unemployed. All the participants who took part in this study had their maternity care delivered by NHS trusts based in the South of England. For a more informed understanding of how COVID-19 affected women in the whole of England it would be necessary to include participants from the Midlands and the North of England. Finally, the interviews were conducted using Microsoft Teams and WhatsApp which required the participants to be computer literate and willing to be involved in an online interview.

## Conclusion

This study assessed the impact of the COVID-19 pandemic on maternity service users’ access to health information. The results suggested that they were affected by poor communication, difficulties in accessing reliable and trustworthy sources of information, inconsistent implementation of government and NHS policy, and were uncertain about taking the COVID-19 vaccine due to limited information provided to them by their responsible NHS trusts. This study is the first to review both maternity service user’s access to health information and decisions around the COVID-19 vaccine. The study contributes to existing literature on the effects of the pandemic on NHS maternity service users and provides areas of consideration for policy and practice going forward. This includes the inclusion of pregnant women and women who are postpartum in vaccine clinical trials to contribute to COVID-19 vaccine acceptability in this population. In pandemic or epidemic response, it is crucial that maternity services are standardised for all service users and that health information distributed by the actors involved is accurate and can be understood by the people it applies to.

## Data Availability

The datasets used and/or analysed during the current study are available from the corresponding author on reasonable request.
